# Comparison of physical fitness and mental health status among school-age children with different sport-specific training frequencies

**DOI:** 10.7717/peerj.10842

**Published:** 2021-02-02

**Authors:** Ruichen Jiang, Chun Xie, Jilong Shi, Xuechen Mao, Qin Huang, Fanying Meng, Zhiguang Ji, Anmin Li, Chunhua Zhang

**Affiliations:** 1 School of Psychology, Shanghai University of Sport, Shanghai, China; 2 School of Teacher Education, Anqing Normal University, Anqing, China; 3 Institute of Physical Education, Huzhou University, Huzhou, China; 4 Shanghai University of Medicine & Health Sciences, Shanghai, China; 5 School of Kinesiology, Shanghai University of Sport, Shanghai, China

**Keywords:** Sport-specific training, Training frequency, Physical fitness, Mental health, Children

## Abstract

This cross-sectional study compared the physical fitness and mental health status of 140 school-age children who participated in sport-specific training with 180 age-matched peers. All the participants were grouped by sport-specific training frequencies in extracurricular time into the following: (i) high sports training frequency group (HFG): training three to five times per week (*n* = 77, mean [SD] age: 9.60 [0.12] years); (ii) low sports training frequency group (LFG): training once per week (*n* = 63, mean [SD] age: 9.88 [0.14] years); and (iii) control group (CG): maintaining routine life (*n* = 180, mean (SD) age: 9.77(0.09) years). Physical fitness status, including body composition (body mass index), endurance (vital capacity; 50 × 8 round trip), speed and agility (50 m sprint), flexibility (sit-and-reach), coordination (1-min rope skipping), and core strength (1-min sit-ups) as well as mental health status was measured. Overall, the results showed that Grade 3 to 4 HFG students showed better total physical fitness scores than the LFG and CG students. Grade 2 and 5 participants in the three groups showed no significant difference in the total physical fitness score. Children in HFG performed better in several PF indicators (i.e., cardiopulmonary function, flexibility, core strength, and coordination) than those in LFG and CG, and children in LFG got a higher score than those in CG on a testing item of 1-min rope skipping. The mental health test results showed that HFG performed better than LFG and CG. The results indicated that participating in sport-specific training 3–5 times per week was beneficial for children’s physical and mental health. Additionally, there was a weak and negative correlation between physical fitness and mental health in LFG and CG, while no correlation was found between physical fitness and mental health in HFG.

## Introduction

Sport-specific training may be defined as an intense year-round training program in a single sport, excluding other sports activities (Brad & Stern, 2014; Jayanthi et al., 2015; Malina, 2010). Many people believe that sport-specific training at an early age could develop elite-level abilities sooner, as there is evidence that differences in training and performance between elite and non-elite athletes appear at the age of seven (Ericsson, Krampe & Tesch-Roemer, 1993; Ward et al., 2004). Therefore, many children select one special sport in extracurricular time to strive for the “elite” status (Brad & Stern, 2014). The data show that about 45 million youths in the US participate in sport, and sport specialization at an early age continues to be a popular choice among adolescents and children worldwide (Jayanthi et al., 2011; Malina, 2010; Merkel, 2013; Metzl, 2002). Studies have revealed that sport-specific training at an early age is beneficial to the physical development of adolescents (Granacher & Borde, 2017; Merkel, 2013). Moreover, direct evidence has also proven the benefits of early sport specialization on the mental health of adolescents and children (Merkel, 2013), and students who participated in sport demonstrated more psycho-social benefits compared to those who were just active in extracurricular programs that were not related to sport (Harrison & Narayan, 2003).

However, other experts suggested that children should participate in a variety of different activities and avoid specialization until puberty (Bergeron et al., 2015). Such recommendations were raised because of some research findings which demonstrated that sport specialization with high-intensity in adolescence may lower proper healthy growth and cause a lot of problems, such as a higher risk of overuse injuries, emotional stress, and burnout (Brad & Stern, 2014; Gould, 2009; Russell & Limle, 2013). Similar results were shown in studies on individuals from diverse sport specialization demonstrating that early sport specialization brought a negative influence on the mental health of adolescents (Dubuc et al., 2010; Martinent et al., 2014; Schmikli et al., 2011).

To sum up, the answer to the question regarding the relationship between early sport specialization and individual health of school-aged children is still unclear and studies on this topic are of great importance (Gould, 2009; Granacher & Borde, 2017; Hallal et al., 2006). The differences between children participating in regular sport-specific training and age-matched peers can be explored for better understanding. The aim of this study was to investigate the physical and mental health of school-age children in China who participated in sport-specific training in extracurricular time by using the physical fitness measurement, psychological measurement, and comprehensive evaluation as the main methods and then compare their results with those of gender-matched peers. This will help the parents and coaches to make reasonable and intentional decisions about whether children should select sport-specific training at an early age. Furthermore, possible associations between participation in sport-specific training and individual health varying with the training frequency were also investigated. Initially, it was hypothesized that (i) children participating in sport-specific training would perform better on physical fitness and mental health measures, (ii) appropriate training frequency will bring about better training effects, and (iii) the level of children’s physical fitness would negatively correlate with their mental health.

## Materials & Methods

### Participants

Three hundred and twenty primary school students from three primary schools in Shanghai city were enrolled to participate in this study. Of 320 students, 140 participated in regular sport-specific training (i.e., tennis, table tennis, and badminton) in extracurricular time, and based on their training frequency, they were divided into two groups: High training frequency group (HFG) and low training frequency group (LFG). HFG consisted of 77 students who attended the sports training for about 3–5 times a week, and the training duration of each time was was 2 h. LFG consisted of 63 students who attended the training for once a week and their training duration was also 2 h. The total training period of the HFG and the LFG was about 20 months. The remaining 180 students were named comparative group (CG), and they did not participate in regular extracurricular sports training activities. The protocol was approved by the Ethics Committee of Shanghai University of Sport (Approval number: 102772019RT073), and all the tests were conducted in accordance with the ethical standards in sports medicine and exercise science (Harriss & Atkinson, 2011). We received written informed consent from all the participants’ guardians.

### Measurements

#### Demographics

Participants’ age, grade, gender, and training frequency were collected using single-item measures.

#### Physical fitness test

Table 1 shows all the indicators used in the physical fitness test. Due to differences in physical characteristics of children at different ages, physical fitness tests differed between different grades. All participants were required to participate in providing two testing items (body mass index and vital capacity). Other items were differentiated according to the grade of the subjects. The total score of physical fitness score was calculated according to the weight derived from the National Student Physical Health Standard of China (MOE of PRC, 2007) (Table 1).

**Table 1:** Physical fitness test and evaluation indicators. Table 1 shows all the indicators used in the physical fitness test. All participants were required to participate in providing two testing items (body mass index and vital capacity). Other items were differentiated according to the grade of the subjects.

**Objects**	**Single index**	**Weight** (%)
Grade two to grade five (Common testing items)	Body mass index	15
	Vital capacity	15
Grade two	50-m sprint	20
	Sit-and-reach	30
	One-minute rope skipping	20
Grade three to grade four	50-m sprint	20
	Sit-and-reach	20
	One-minute rope skipping	20
	One-minute sit ups	10
Grade five	50-m sprint	20
	Sit-and-reach	10
	One-minute rope skipping	10
	One-minute sit ups	20
	50 × 8 round trip	10

#### Mental health test

The mental health level of the students was measured using the Revised Scale of the Diagnostic Test of Unquiet Tendency (Zhou, 1991). The scale consists of eight sub-scales and one validity scale, and there are 100 items in the scale. Each item has only two choices of “yes” and “no”. If the validity scale score is more than 7 points, the participant may be considered to have cheated in the test to obtain good results and the test results would be considered incredible. Cumulative scores for all items ranging from 0 (good mental state) to 65 (poor mental health) were obtained. The mental health of primary and secondary school students has been evaluated using a well-standardized MHT scale, which is reliable and valid, as shown in various studies (Liu, Yuan & Li, 2013; Xu, Mu & Xie, 2015). Cronbach’s alpha coefficient, split-half reliability, and retest reliability of the MHT scale are between 0.81 and 0.89.

### Procedure

This study involved a combination of purposive sampling and random sampling. Participants in the HFG and LFG were recruited via purposive sampling, while the participants in the CG were recruited via random sampling. The headmasters of selected schools helped in deciding a specific date for assessment, and measurements were embedded in the physical education schedule to facilitate participant access and tracking. One group of testers collected physical fitness data, and another group carried out the psychological survey and collected data. All the participants underwent these two tests, and they were scheduled for the same time of the day (always from 8 am to 11 am). Before conducting the physical fitness test, a thorough investigation of the participating school was done to ensure that they meet the requirements. The testers were trained strictly in accordance with the requirements of the National Physical Fitness Test (primary school group). Physical fitness and mental health test were carried out in the outdoor field and indoor gymnastics, respectively.

### Data analyses

All the data analyses were performed using SPSS 21 (IBM SPSS Statistics, Version 21, 2012). In this study, missing data (<3%) were treated as incorrect answers (Griffiths et al., 2004). Differences between means of different populations were analyzed using one-way covariance analysis adjusted for age and gender. If *p* < 0.05, the results was considered statistically significant. In case if variances were not homogeneous, Mann–Whitney *U* tests (test of mean ranks; *p* < 0.05) were conducted to evaluate group differences, and summary scores were reported as mean (SE). Pearson correlation analysis was used to explore the connection between physical fitness and mental health in the three groups. In the analyses, the Bonferroni correction was used for multiple comparisons.

## Results

### Demographic characteristics

Table 2 presents the participant demographic characteristics. Of 320 students who took part in the study, 306 (83.33%) completed both tests successfully. The age range of participants was 7–13 years, with 9.76 years (SE = 0.06) being the mean age.

**Table 2:** Demographic characteristics of the study participants (M ± SE).

**Characteristics**		**HFG (*n* = 74)**	**LFG (*n* = 60)**	**CG (*n* = 172)**
Age (years)		9.59(0.13)	9.92(0.15)	9.77(0.09)
Height (cm)		138.92(0.92)	140.92(1.24)	140.80(0.76)
Gender (Number)	Male	31(20.80%)	32(21.48%)	86(57.72%)
	Female	43(27.39%)	28(17.83%)	86(54.78%)
Grade (Number)	Two	20(25.32%)	14(17.72%)	45(56.96%)
	Three	19(24.36%)	15(19.23%)	44(56.41%)
	Four	19(24.05%)	16(20.25%)	44(55.70%)
	Five	16(22.86%)	15(21.43%)	39(55.71%)

### Physical fitness status

**Figure 1:**
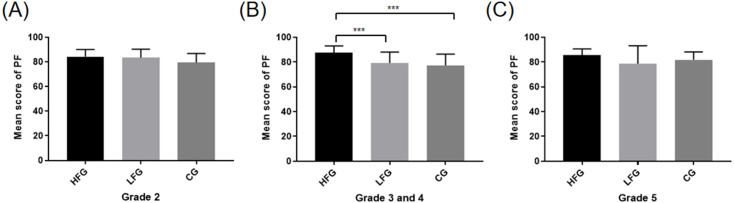
Mean score for total physical fitness of different grades in three groups. (A) Grade 2, (B) Grade 3 and 4, (C) Grade 5. The *x*-axis indicates three groups with different training frequencies. The *y*-axis indicates mean score of physical fitness (PF). The error bar indicates the standard deviation (SD). ***Significant difference between the two groups, *p* < 0.001.

The between-subject factor of training frequency was analyzed using one-way covariance analysis adjusted for age and gender, and the impact on physical fitness was explored. The effect of training frequency showed significance (*F* (1,306) = 20.59, *p* < 0.001). The results showed that from grade 3 to grade 4, HFG got a higher score than LFG and CG. However, there were no significant differences between these groups in Grade 2 and Grade 5. Figure 1 shows the results. The different results may be related to different grades. For the students in grade two, due to their limited physical activity, it may take more time of them to learn proper posture to grip the rackets and stroke the ball, while not much time was really spent on training. The participants in grade five were unable to practice for a longer time due to the high school entrance examination. As a result, there was no significant difference among the three groups in grade 2 and 5.

**Figure 2:**
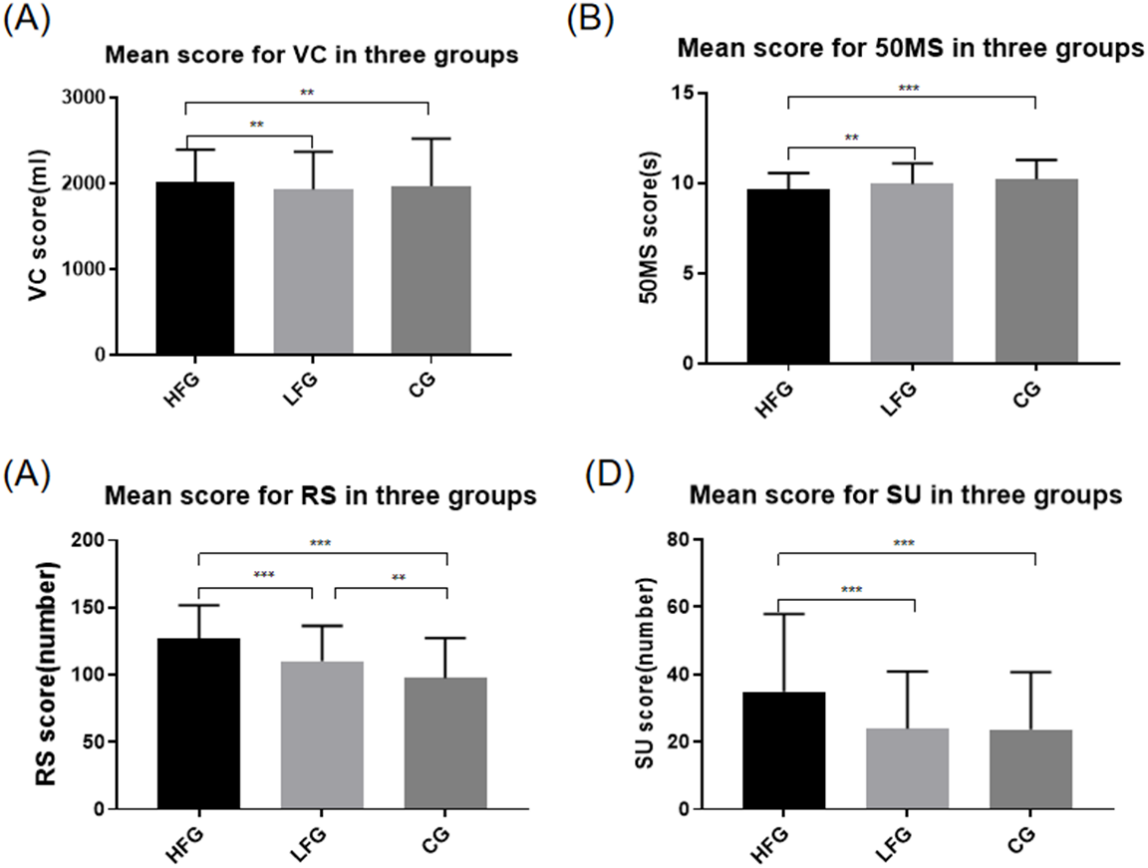
Comparison of the item score in three groups. (A) Vital capacity, (B) 50 m sprint, (C) 1-min rope skipping, (D) 1-min sit-ups. The *x*-axis indicates three groups with different training frequencies. The *y*-axis indicates mean score of each testing item. The error bar indicates the standard deviation (SD). **Significant difference between the two groups, *p* < 0.01. ***Significant difference between the two groups, *p* < 0.001.

Figure 2 shows the distribution of physical fitness characteristics in the three groups. No differences in terms of SAR and 50 × 8 RT among these groups (*p* > 0.05) were observed. However, significant differences among VC, 50MS, RS, and SU were found, which showed that scores of HFG were better (*p* < 0.01) than those of LFG and CG and indicated that students in HFG performed better on cardiopulmonary function, flexibility, body coordination, and core strength. Additionally, LFG got a better score than CG on RS item (*p* < 0.01).

### Mental health status

**Figure 3:**
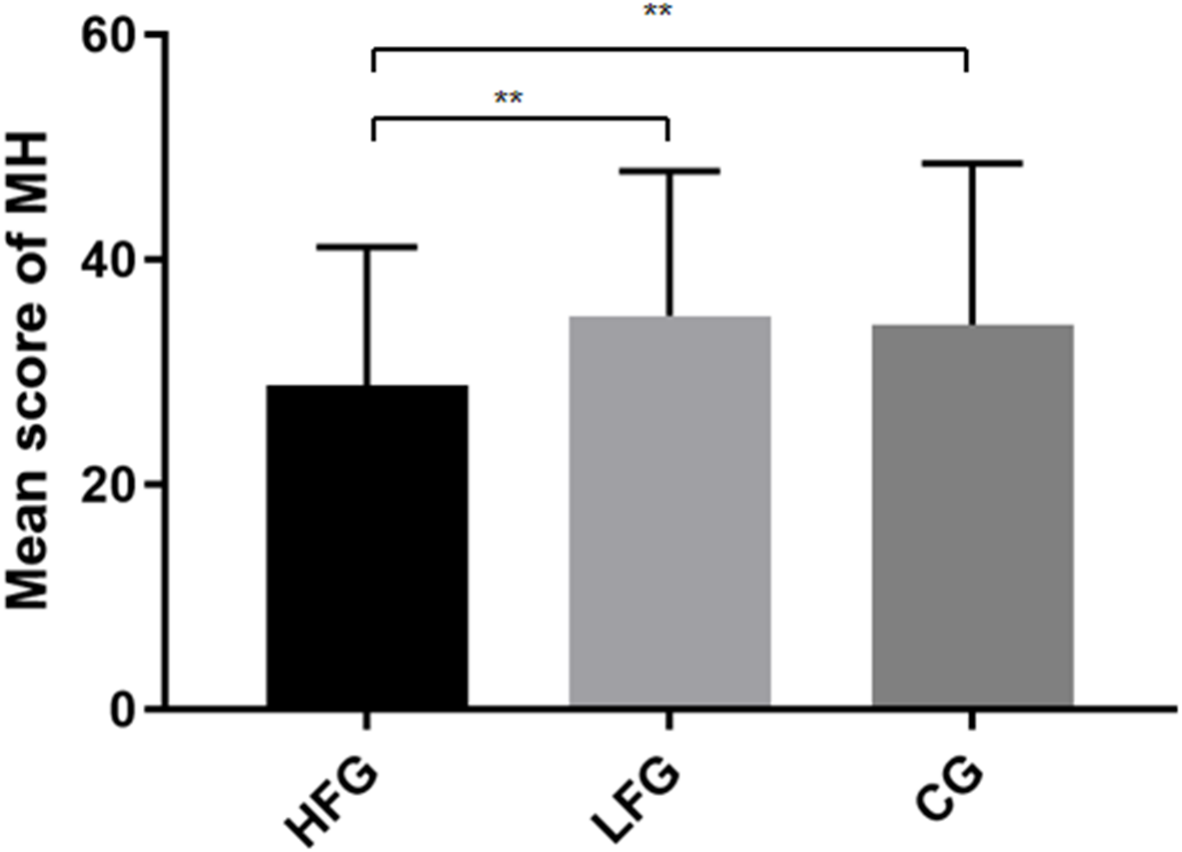
Mean score for mental health in three groups. The *x*-axis indicates three groups with different training frequencies. The *y*-axis indicates the mean score of mental health (MH). The error bar indicates the standard deviation (SD). **Significant difference between the two groups, *p* < 0.01.

The between-subject factor of training frequency was analyzed using one-way covariance analysis adjusted for age and gender, and its impact on mental health was explored. Training frequency showed a significant effect on mental health (*F* (1,306) = 6.07, *p* < 0.01). Figure 3 shows the results.

Significant differences were observed in terms of mental health (*p* < 0.01). The scores of HFG were better than that of LFG and CG on mental health (35.02 > 28.85, *p* < 0.01; 34.20 > 28.85, *p* < 0.01) status. This outcome was consistent with previous studies and indicated that regular sports training could benefit children’s psychological development.

### Correlation of physical fitness variables with mental health

The theory of unity of body and mind holds that improvement of physical fitness contributes to the development of mental function. To examine the associations between physical fitness and mental health in the three groups, we conducted a correlation analysis. There was a weak and negative correlation between physical fitness and mental health in LFG (*r* =  − 0.257, *p* < 0.05) and CG (*r* =  − 0.220, *p* < 0.01), but no correlation was found between physical fitness and mental health in HFG (*p* > 0.05).

## Discussion

The results of this study showed differences in the physical and mental health of children who participated in sport specialization with different training frequencies. In general, the physical fitness and mental health scores of children in the HFG were better than those of the other children.

Specifically speaking, grade 3–4 HFG students showed higher total PF scores than LFG and CG students, and these results were similar to the previous research conclusions (Drenowatz et al., 2013; Golle et al., 2014). However, there was no significant difference in the total PF scores among the second-grade students with different training frequencies. This could be attributed to the total amount of time spent in sports training. Due to the limited physical activity of students in grade two, it may take more time for them in learning proper posture but not actual training. No significant difference was observed in the total PF scores among the fifth-grade students with different training frequencies, possibly due to the High School Entrance Examination because of which there was not enough time for the grade 5 students to participate in specific training after classes.

The physical fitness of school-age children with different training frequencies was not only reflected in the total score, but also in the factor score, such as VC, 50MS, RS, and SU. As one of the important functional indices for evaluating the level of human growth, VC is the maximum amount of air a person can expel from the lungs after a maximum inspiration. The result revealed that frequent and regular physical exercise may help to enhance the individual’s cardiopulmonary function. In this study, HFG students showed better performance at 50MS, which indicated that regular sports training could benefit the speed and agility of children. Since the subjects of this study participate in confrontational events and the important characteristic of these events is that participants have to deal with emergencies on the field and make quick position changes and decisions (Ozmen & Aydogmus, 2016). Thus the HFG students could score better at 50MS after a long period of training. HFG also reported a higher score than LFG and CG on the RS test, which showed a better ability of body coordination, while LFG also showed better performance than CG on this testing item. These results indicated that participating in low-frequency sport-specific training could also effectively improve an individual’s balancing ability. Students with improved sensitive quality and body coordination could perform effectively in the field. The scores of HFG on SU were also better, which indicated better core strength (Tiwari, Rai & Srinet, 2016). It is known that the “core area” strength maintains the stability of body balance during rapid movements and prevents lower extremities injuries (Ozmen & Aydogmus, 2016; Zazulak et al., 2007), so this ability is very important for participants in confrontational events and could get better with months of training.

Prior studies have suggested that psychological performances would decline among adolescents due to sport-specific demands (e.g., high training volumes, a large number of competitions) (Kusurkar et al., 2012; Malina, 2010; Wagner et al., 2015). Conversely, this study revealed outcomes showing a positive effect of physical activity on mental health among children participating in sport-specific training, and this difference might be contributing to the effective control of training time or training intensity (Farrally et al., 2003; Malina & Cumming, 2004; Snyder et al., 2010). Psychological benefits could gained when training intensity was well controlled. Sport specialization plays an important role in mental health in three ways: social interaction in sports, various positive emotional experiences gained in sports, and related physiological changes (Breslin et al., 2017; Li, 2015). The social aspect of sports may be the key factor for positive mental health outcomes among children and adolescents. Students participating in sport-specific training usually have a more intimate relationship with their classmates since they always play together, promising them stronger psychological support than other students. In the process of sports training, individuals are more likely to experience positive emotional experiences, and they are also more likely to infect others with their own emotions. These good interpersonal relationship and positive emotional experience will eventually lead to the transformation of individual mental state.

The comparative study among the three groups with different training frequencies revealed that the regular training of 3–5 times a week has a good overall improvement in students’ health. However, it is not true that more training time would result in a better effect. A clear correlation between training volume, intensity, and injury risk, particularly overuse injuries (Jayanthi et al., 2011; Sarah, Emery & Meeuwisse, 2008), was reported. Children and youth aged 5–17 are advised to undergo at least 1 hour of moderate-to vigorous-intensity physical activity (MVPA) a day at a frequency of 3 times per week to gain maximum exercise benefit, according to World Health Organization (WHO) recommendation (World Health Organization, 2010).

In this study, a weak correlation between physical fitness and mental health was observed in CG and LFG, while no correlation was found between physical fitness and mental health in HFG. These findings were consistent with the previous studies (Hussain et al., 2013; Regehr, Glancy & Pitts, 2013). The reason for this phenomenon might be that sport training does not have a synchronous effect on physical and mental health. For normal population and individuals with moderate exercise, there were more or less differences in physical fitness or mental health among different individuals, thus their physical fitness and mental health scores were obviously correlated. While for students in the HFG, the physical fitness and mental health both reached a high level, and eventually there was little difference between different individuals, thus no significant correlation between physical fitness and mental health was found.

Despite the benefits of early sport specialization in health improvement, limitations of the study warrant mention. First, Our test was a cross-sectional test. Since it is difficult to determine whether baseline level differences existed among these groups, longitudinal studies are needed in the future to explore the impact of sport-specific training on the physical and mental health of children. Secondly, the relationship between sport-specific training and individual health may be affected by the type of sports. Further research, if possible, could take this into account. Finally, there were differences in physical fitness and mental health among students at different grades, but whether these differences would change with age should be investigated by continuous follow-up studies.

## Conclusions

To summarize, the results of this study showed that regular and sport-specific training of about 3–5 times per week has a profound effect on children’s physical fitness and mental health. The complexities of the interrelations between sport-specific training and individual health among school-age children were highlighted in this study. This study also supported the importance of early sport specialization in developing physical fitness and mental health.

##  Supplemental Information

10.7717/peerj.10842/supp-1 Data S1 Raw data of the study subjects

## Additional Information and Declarations

### Data Availability

The following information was supplied regarding data availability: The raw measurements are available as Supplemental File.
